# Characterization of *Pterotylenchus cecidogenus* in *Desmodium ovalifolium* cover crop from oil palm plantations in central Colombia

**DOI:** 10.21307/jofnem-2021-099

**Published:** 2021-12-01

**Authors:** Oscar Velandia, Yuri Mestizo, Héctor Camilo Medina, Donald Riascos-Ortiz, Francia Varón De Agudelo, Greicy Andrea Sarria

**Affiliations:** 1 Pests and Diseases Program, Cenipalma. Experimental Field Palmar de La Vizcaína, Km 132 Vía Puerto Araujo-La Lizama, Barrancabermeja, Santander, 111611, Colombia; 2 Facultad de Agronomía de la Universidad del Pacífico, Buenaventura, Valle del Cauca, Campus universitario, Km 13 vía al aeropuerto, Barrio el Triunfo

**Keywords:** Diagnostic, Molecular biology, Phylogenetic analysis, *Pterotylenchus cecidogenus*, Stem gall

## Abstract

Until recently, the stem gall nematode *Pterotylenchus cecidogenus* was only registered in eastern Colombia. However, the disease has recently been observed in central Colombian oil palm plantations that use *Desmodium ovalifolium* as a cover crop. Soil, root, stem, and leaf samples were collected from *D. ovalifolium*. Plants showed foliar yellowing, leaf drying, and galls within stem nodes. Nematodes were identified, and the distribution, population density, and relative importance of different genera were determined. We performed morphometric and molecular identification of nematodes associated with gall symptoms. The D2-D3 segment of the large subunit-28S of ribosomal ribonucleic acid (RNA) and internal transcribed spacer (ITS) was sequenced, and phylogenetic analysis was performed. *P. cecidogenus* mainly occurred in the galls and to a lesser extent in the roots and soil. Nematodes were not found in leaf or inflorescence tissue. Morphological and morphometric data confirm the presence of *P. cecidogenus* in the stems of *D. ovalifolium* with gall symptoms. This study is the first to report deoxyribonucleic acid (DNA) sequences of *P. cecidogenus*. Based on D2-D3 and ITS partial sequences, *P. cecidogenus* is a sister species of the leaf-galling nematode *Ditylenchus phyllobius* (Sinm. *Orrina phyllobia*).

Colombia is the fourth largest palm oil producer in the world and the first in Latin America. The country has more than 535,000 hectares planted in 112 towns across 20 states, making palm oil one of the main national agricultural sectors (Fedepalma, 2018; SISPA 2019). However, Colombian oil palm production is affected by two lethal diseases (sudden wilt and lethal wilt). In both cases, the primary management strategy is to eliminate grasses and establish legume cover crops (Arango et al., 2011; Sierra et al., 2014).

Legume cover crops are widespread and are considered essential components of productive systems, including oil palm, rubber, coffee, and bananas (Baligar et al., 2006). For oil palm, the cover is established in the immature stage of the crop, during which palm foliage cannot protect the soil from solar radiation, wind, and erosion (Ruíz and Molina, 2014). D. *heterocarpon* (L.) DC. (Fabaceae) (cv. Maquenque) is commonly used in palm oil plantations; alternatively, *Desmodium ovalifolium* is sometimes favored owing to of its covering capacity and resistance to shading. However, on the eastern Colombian plains, *D. ovalifolium* is affected by the stem gall nematode *Pterotylenchus cecidogenus* (Varón et al., 2017).

The first symptoms associated with *P. cecidogenus* in *Desmodium* are shoot leaves with chlorosis, which later wilt and finally causes necrosis and defoliation. The stems and shoots of plants with these symptoms host galls located at the nodes. Young galls are small and light brown; however, they form a subberous that cracks and dries out, turning the gall a dark, almost black color. The galls cannot detach without damaging the stem. The nematode destroys the stem’s cortical and vascular tissues, causing the death of the plant (Lenné, 1983; Lehman, 1991; Varón et al., 2017).

The nematode does not inhibit *Desmodium* seed germination but reduces plant survival and root and stem growth (Stanton, 1986). Gall formation is related to the nematode population and age of the plant. In Stanton (1986), the nematode population increased 100 times 52 days after plants were inoculated. Although the nematode does not need wounds to penetrate the stem, injuries caused by mechanical damage are quickly colonized by the parasite (Lehman, 1991). This nematode’s life cycle takes approximately 2 weeks and presents four juvenile stages (the first occurring inside the egg) for female members; thus far, male members have not been identified (Stanton, 1990). In addition, Stanton (1990) found that *P. cecidogenus* moves faster from dead tissue than within tissues of *D. ovalifolium* plants, and that nematodes probably move from one site to another by a film of water outside the stem. Nematodes move very little in the soil, and so it is difficult to find them in soil samples.

The stem gall nematode *P. cecidogenus* was first identified as a new genus and species belonging to the Anguinidae family in 1981, affecting *Desmodium* from Carimagua in the Llanos Orientales of Colombia. In the original description, morphological and morphometric data were reported for *P. cecidogenus* (Siddiqi and Lenné, 1984). However, thus far, no molecular data are reported for this nematode in worldwide databases; therefore, its evolutionary relationships based on deoxyribonucleic acid (DNA) sequences remain unknown.

The cultivation of oil palm is a growing industry in the Colombian agricultural sector. Until recently, *P. cecidogenus* was only registered in eastern Colombia. However, the disease has recently been observed in central Colombian oil palm plantations that use *Desmodium ovalifolium* as a cover crop. The *D. ovalifolium* is affected by yellowing and subsequent drying of the plants, causing losses for palm growers. As such, timely management strategies to prevent the dissemination and/or establishment of the disease to other plantations is needed. Thus, the present study had the following objectives: (i) to verify the presence of *P. cecidogenus* in *D. ovalifolium* from the central region of Colombia, (ii) confirm the taxonomic identity of *P. cecidogenus* through morphological, morphometric, and molecular analyses, and (iii) determine the phylogenetic relationships of *P. cecidogenus* through molecular data.

## Materials and methods

### Symptom observation and sampling

We visited 30 oil palm plantations (*Elaeis guineensis*) of between 3 and 6 years of establishment with *Desmodium ovalifolium* as a cover crop. To verify the health status of the cover crop and identify plants with abnormal aspects, one sample per lot was taken, each of which was composed of five subsamples (Table 1). The selected plantations were located in central Colombia (departments of Santander [26], Norte de Santander [1], Cesar [3]).

**Table 1.** Locations of plantations sampled for the recognition of nematodes in *Desmodium ovalifolium.*

Sample No.	Coordinate Length	Latitude	Location	Oil palm planting year	Presence of galls in *D. ovalifolium*
1	6,7073	‒74,0007	Puerto Parra (Santander)	2013	+
2	6,7073	‒73,9994	Puerto Parra (Santander)	2012	+
3	6,7722	‒74,0502	Puerto Parra (Santander)	2013	+
4	6,9736	‒73,6815	Barrancabermeja (Santander)	2012	+
5	6,9066	‒73,6816	San Vicente de Chucurí (Santander)	2007	+
6	6,9823	‒73,6232	San Vicente de Chucurí (Santander)	2004	+
7	7,0328	‒73,5576	San Vicente de Chucurí (Santander)	2010	‒
8	7,2072	‒73,5793	Puerto Wilches (Santander)	2013	+
9	7,2636	‒73,5804	Rio Negro (Santander)	2012	‒
10	7,6562	‒73,5765	Rio Negro (Santander)	2012	‒
11	7,2308	‒73,5588	Sabana de Torres (Santander)	2001	+
12	7,1554	‒73,5183	Girón (Santander)	2014	‒
13	7,1655	‒73,5009	Girón (Santander)	2013	‒
14	7,3257	‒73,5661	Sabana de Torres (Santander)	2007	‒
15	7,3862	‒73,5256	Sabana de Torres (Santander)	2013	+
16	6,7806	‒73,9080	Simacota (Santander)	2010	‒
17	6,7781	‒73,9042	Simacota (Santander)	2010	‒
18	6,7923	‒73,7590	Simacota (Santander)	1999	+
19	7,3116	‒73,8327	Puerto Wilches (Santander)	2014	+
20	7,3000	‒73,8793	Puerto Wilches (Santander)	2010	‒
21	7,2451	‒73,8438	Puerto Wilches (Santander)	1997	+
22	7,2836	‒73,7143	Puerto Wilches (Santander)	2006	+
23	7,3301	‒73,6687	Puerto Wilches (Santander)	2009	+
24	7,2260	‒73,5466	Sabana de Torres (Santander)	2013	‒
25	7,2340	‒73,5445	Sabana de Torres (Santander)	2013	‒
26	7,7864	‒73,4530	San Alberto (Cesar)	2008	‒
27	7,7864	‒73,4538	San Alberto (Cesar)	2009	‒
28	7,6551	‒73,3853	La Esperanza (Norte de Santander)	2008	+
29	7,7678	‒73,4023	San Alberto (Cesar)	2005	+
30	7,2159	‒73,5777	Sabana de Torres (Santander)	2008	+

Note: +presence of galls, ‒ absence of galls.

Symptomatic tissue samples as stems (with or without galls), leaves, pods, and inflorescences were collected and kept in labeled plastic bags and refrigerated until processing. Simultaneously, at locations where plants with symptoms were found, samples of young roots and rhizospheric soil were collected from close to the area with the highest concentration of roots in order to determine if the nematode affected the root system or survived in the ground.

### Sample processing

Nematodes were extracted using the oxygenation-decantation method (Ravichandra, 2014). In brief, 1 g of fresh tissue from each of the sampled organs was cut into small portions and placed in a decantation sieve without a paper towel but with enough water to cover the sample. After 24 h, the decantation plate with the nematode suspension was removed and concentrated to 20 mL with a 400-mesh sieve (Varón de Agudelo and Castillo, 2001).

Soil nematodes were extracted by suspension, filtration, and decantation methods (Ravichandra, 2014). In brief, 100 cm^3^ of soil was placed in a container with water. After stirring for 2 min, the suspension was passed through a series of three sieves arranged from larger to smaller mesh diameters (reference No. 20 = 840  µm, 200 = 75  µm, 400 = 37  µm). The contents of the last two sieves were collected for decantation using a sieve previously arranged with a paper towel, and rested on a decantation plate with sufficient water. After 24 hr, the nematode suspension contained in the decantation plate was removed and concentrated to 20 mL with a 400-mesh sieve (Varón de Agudelo and Castillo, 2001).

### Morphological and morphometric identification of the nematode

For the morphological and morphometric identification of *Pterotylenchus*, 23 females were treated with heat at 60°C for 4 min and fixed in 2% formalin. Then, semipermanent preparations were performed, and morphometric data (Table 2) were registered following Siddiqi and Lenné (1984). The morphometric data were obtained using a compound microscope (ZEISS Axio A1, Suzhou, China).

**Table 2.** Morphometric data for *Pterotylenchus cecidogenus*, including those characterized in the present study, those reported in the original description, and those from reference populations of *Orrina phyllobia* (=*Ditylenchus phyllobius*).

Measurement	Pterotylenchus cecidogenus (females)^1^ n = 23	Pterotylenchus cecidogenus (females)^2^ n = 30	Orrina phyllobia (females)^3^ n = 20	Ditylenchus phyllobius(females)^4^ n = not available	Ditylenchus phyllobius(females)^5^ n = not available
Body length	708.4 ± 36.5 (646.4–790.3)	640 (590–800)	696.2 (637–785)	(590–840)	684 (592–838)
*a*	30.5 ± 1.8 (26.5–33.7)	28 (22–35)	31.6 (22.5–39)	(20–32)	25 (20–32)
*c*	12.5 ± 1.6 (11.1–17.4)	11.0 (9.6–12.5)	18.3 (17–19.6)	(11.4–17.6)	14.6 (11.4–17.6)
*c*´	4.2 ± 0.4 (2.8–4.8)	4.5 (3.6–5.2)	4.4 (3.5–5.6)	(2.9–4.5)	3.7 (2.9–4.5)
V%	82 ± 0.9 (80.0–84.3)	82 (80.0–84.0)	79.2 (78–81)	(78–85)	81 (78–84)
Stylet	9.1 ± 2.4 (5.2–11.9)	9.5 (8.0–11.0)	9 (8–9)	(9–10)	(9–11)
Max. body diameter	23.2 ± 1.3 (21.0–26.6)	22 (20.0–25.0)	-	-	-
Anal body diameter	13.5 ± 1.0 (11.8–14.9)	-	-	-	-
Tail length	57,6 ± 6.1 (40.4–64.1)	60 (53.0–68.0)	-	-	-

Note: *L* = total body length, *a* = total body length divided by maximum body diameter, *c* = total body length divided by tail length, *c*´ = tail length divided by diameter at the anal aperture, V% = position of vulva from anterior end expressed as percentage of body length. Present study of Colombian central zone. Carimagua, Colombia (Siddiqi and Lenné, 1984). Guanajuato, Mexico (Medina et al., 2016). Nickle (1991). Brzeski (1991).

### Molecular identification

DNA extraction was performed using the proteinase K method (Riascos-Ortiz et al., 2019). In brief, the nematodes were divided into three parts with a sterile scalpel and transferred to Eppendorf tubes with 15 µL lysis buffer (50 mM KCl, 10 mM Tris pH 8.0, 15 mM MgCl2, 0.5% Triton × 100, 4.5% Tween 20, and 0.09% Proteinase K). Subsequently, the tubes were incubated at ‒80°C for 15 min, 65 °C for 1 h, and 95°C for 15 min, centrifuged at 16,000 × g for 1 min, and stored at ‒20°C. The polymerase chain reaction (PCR) amplification of the expansion segment D2-D3 of the large subunit of ribosomal DNA (28S) was performed with the primers D2A (5′-ACAAGTACCGTGAGGGAAAGTTG-3′) forward and D3B (5′-TCCTCGGAAGGAACCAGCTACTA-3′) reverse, according to De Ley et al. (1999). In addition, the partial region of the internal transcribed spacer (ITS), which includes ITS1, 5.8S, and ITS2, was amplified using the primers TW81 forward (5′-GTTTCCGTAGGTGAACCTGC-3′) and AB28 reverse (5′-ATATGCTTAAGTTCAGCGGGT-3′), as proposed by Maafi et al. (2003). The PCR conditions for the amplification of both partial regions were initial denaturation for 2  min at 94°C, followed by 40 cycles of 45 s at 94°C, 45 s at 55°C, 1 min at 72°C, and a final extension of 10 min at 72°C. A total of 25 PCR products were sequenced in both directions by Bionner (South Korea).

### Phylogenetic analysis

The consensus sequences obtained (12 of D2–D3 and 13 of ITS) were edited using the Geneious software (Kearse et al., 2012). Once the sequence editions were carried out, their identities were confirmed using the BLASTn software (http://www.ncbi.nlm.nih.gov/BLAST). Subsequently, the sequences presented under the accession numbers in Table 3 were independently aligned and analyzed using the MUSCLE algorithm included in the MEGA6 program (Tamura et al., 2013). Based on both obtained matrices, the nucleotide substitution models were determined by taking into account the Bayesian information criterion (BIC) using the ModelGenerator v.0.851 software (Keane et al., 2006). The phylogenetic relationships based on D2–D3 and ITS were determined by the maximum likelihood (ML) method based on the Tamura-Nei model (Tamura et al., 2013), which was used to model the differences in evolutionary speed between locations. Internal support of the nodes was performed using the bootstrap method with 1000 replicates. *The Cervidellus cervus* sequence was used as an external group (HM452377) for D2-D3 and *Radopholus similis* (GQ281456) for ITS.

**Table 3.** Information of sequences D2-D3 and internal transcribed spacer (ITS) of ribosomal deoxyribonucleic acid (DNA) downloaded from GenBank and obtained in the present study.

Species name	Location	Host plant	D2-D3 accession number	ITSaccession number	Reference or source
*P. cecidogenus*	Colombia	*D. ovalifolium*	MW208689; MW208690; MZ404621; MZ404622; MZ404623; MZ404624; MZ404625; MZ404626; MZ404627; MZ404628; MZ404629; MZ404630	MZ449098;MZ449099;MZ449100;MZ449101;MZ449102;MZ449103;MZ449104;MZ449105;MZ449106;MZ449107;MZ449108;MZ449109;MZ449110	Present study
*Anguina tritici*	China	*Triticum* sp.	DQ328723; KC818620	-	Subbotin et al. (2006)
*Anguina graminis*	Russia	*Festuca rubra*	-	AF396351	Subbotin et al. (2004)
*Anguina wevelli*	USA	*Eragrostis curvula*	-	AM888393; KU052862	Song et al. (2015)
*Anguina amsinckiae*	USA	*Amsinckia* sp.	-	MK032870	Cid del Prado Vera et al. (2018)
*Subanguina chilensis*	Chile	-	DQ328724	-	Subbotin et al. (2006)
*Subanguina moxae*	China	*Artemisia argyi*	JN885540	-	Yao et al. (2012)
*Subanguina radicicola*	Belgium; China	*Poa* sp.	DQ328721	AF396365; JN885538	(Subbotin et al., 2004; Subbotin et al., 2006)
*Subanguina danthoniae*	USA	*Danthonia californica*	-	MK032869	Cid del Prado Vera et al. (2018)
*Heteroanguina graminophila*	Russia	*Calamagrostis* spp.	DQ328720	AF396315; AF396318	(Subbotin et al., 2004; Subbotin et al., 2006)
*Mesoanguina millefolii*	Russia	-	DQ328722	-	Subbotin et al. (2006)
*Ditylenchus destructor*	Russia; Poland; China; Iran	*Solanum tuberosum*; *Ipomoea batatas*	DQ328727; EU400639; HQ235698	KC923223; KC923224	(Subbotin et al., 2006; Subbotin et al., 2011; Jeszke et al., 2013; Mahmoudi et al., 2020)
*Ditylenchus dipsaci*	Yemen; Mexico	*Allium sativum; Medicago sativa*	JF327759	KY348764	Rosas-Hernández et al. (2017)
*Ditylenchus gallaeformans*	Brazil	*Miconia albicans; Miconia coralline; Leandra lacunosa*	JQ429769; JQ429770	JQ429778; JQ429779	Oliveira et al. (2013)
*Ditylenchus drepanocercus*	Brazil	*Miconia calvescens*	JQ429772	-	Oliveira et al. (2013)
*Ditylenchus oncogenus*	Italy	*Sonchus bulbosus*	KF612015	-	Vovlas et al. (2015)
*Ditylenchus phyllobius*	Mexico	*Solanum elaeagnifolium*	KT192617, KT192618	KT192615; KT192616	Medina et al. (2016)
*Ditylenchus persicus*	Iran	*Vitis vinifera*	KX463285	KX463286	Esmaeili et al. (2017)
*Ditylenchus weischeri*	Canada	*Cirsium arvense*	MG551902	MG386845	Madani and Tenuta (2018)
*Ditylenchus gigas*	Italy; Iran	*Vicia faba; Allium sativum*	HQ219216	KJ653270	Vovlas et al. (2011)
*Ditylenchus arachis*	China	*Arachis hypogaea*	KX426054	JN635037; JX040545	Zhang et al. (2014)
*Ditylenchus halictus*	Germany	*Halictus sexcinctus*	AY589364	-	Ye et al. (2007)
*Ditylenchus gilanicus*	Iran	*Fagus orientalis*	MG742325	-	Yaghoubi et al. (2018)
*Cervidellus cervus*	USA	*Ferocactus*	HM452377	-	Bostrom et al., (2011)
*Radopholus similis*	Colombia	*Musa* sp.	-	GQ281456	Múnera et al. (2010)

### Quantification and analysis of nematode populations

To quantify the nematode populations present in 1 gram of fresh tissue in each sample, three aliquots of 1  mL were taken and counted in a chamber under a light microscope (Olympus PX40, Allentown, PA) and an adapted camera (Olympus DP 73, Allentown, PA). The same methodology was used to quantify the nematode population in 100 cm^3^ of soil from each sample. For the analysis of the populations of nematodes present in the soil and tissue of *Desmodium* plants, parasitic and ecological parameters such as frequencies and prominence values were taken into consideration, including (Norton, 1978; Volcy, 1998):

Absolute frequency = (number of samples in which a genus appears/total samples evaluated) * 100;

Relative frequency = (absolute frequency of the nematode/sum of absolute frequencies) * 100;

Absolute density = number average of individuals per 100 g of soil or number of individuals per 1 g of tissue;

Relative density = (absolute density of the nematode genus/sum of the absolute densities of all genera) × 100;

Prominence value = absolute density * √ absolute frequency;

Relative prominence value = (genus prominence value/sum of the prominence value of all genera) × 100.

## Results

### Description of symptoms

We collected 30 samples from 30 plots located on 28 palm oil plantations in the departments of Cesar (26), Santander (3), and Norte de Santander (1), all of which utilized *Desmodium ovalifolium* as the cover crop (Table 1).

**Figure 1:**
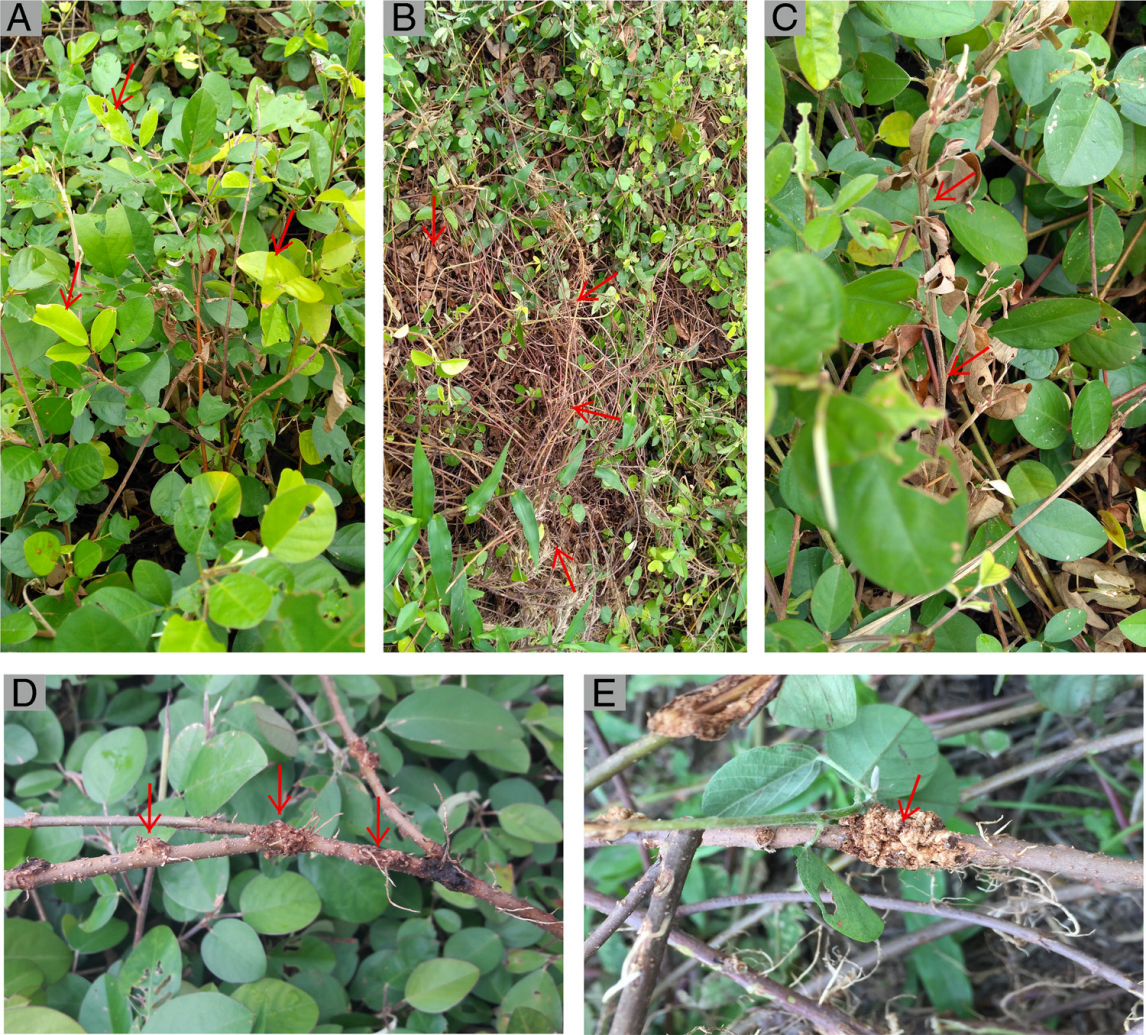
Symptoms caused by *Pterotylenchus cecidogenus* nematode on *Desmodium ovalifolium*. (A) Chlorosis on leaves. (B) Patches caused by drying of plants. (C) Drying and death of the plant. (D) Galls at stem nodes. (E) Old and cracking galls, and the affected cortical tissue. All symptoms are indicated by arrows.

The symptoms observed in diseased plants of *D. ovalifolium* were characterized by yellowing, chlorosis, drying of leaves, wilting of shoots, and death of branches and plants (Fig. 1). In the stem, galls were observed in the nodes, more frequently in the basal part of the stems, and in some cases in the upper nodes. In the initial stages, galls were light brown in color; as they became older, galls became dark brown. The oldest galls presented a cracked suberous, which, when dried, acquired a dark, almost black color. The galls were an integral part of the stem, and it was not possible to separate them without damaging the tissue (Fig. 1E).

### Morphological and morphometric identification of the nematode

**Figure 2:**
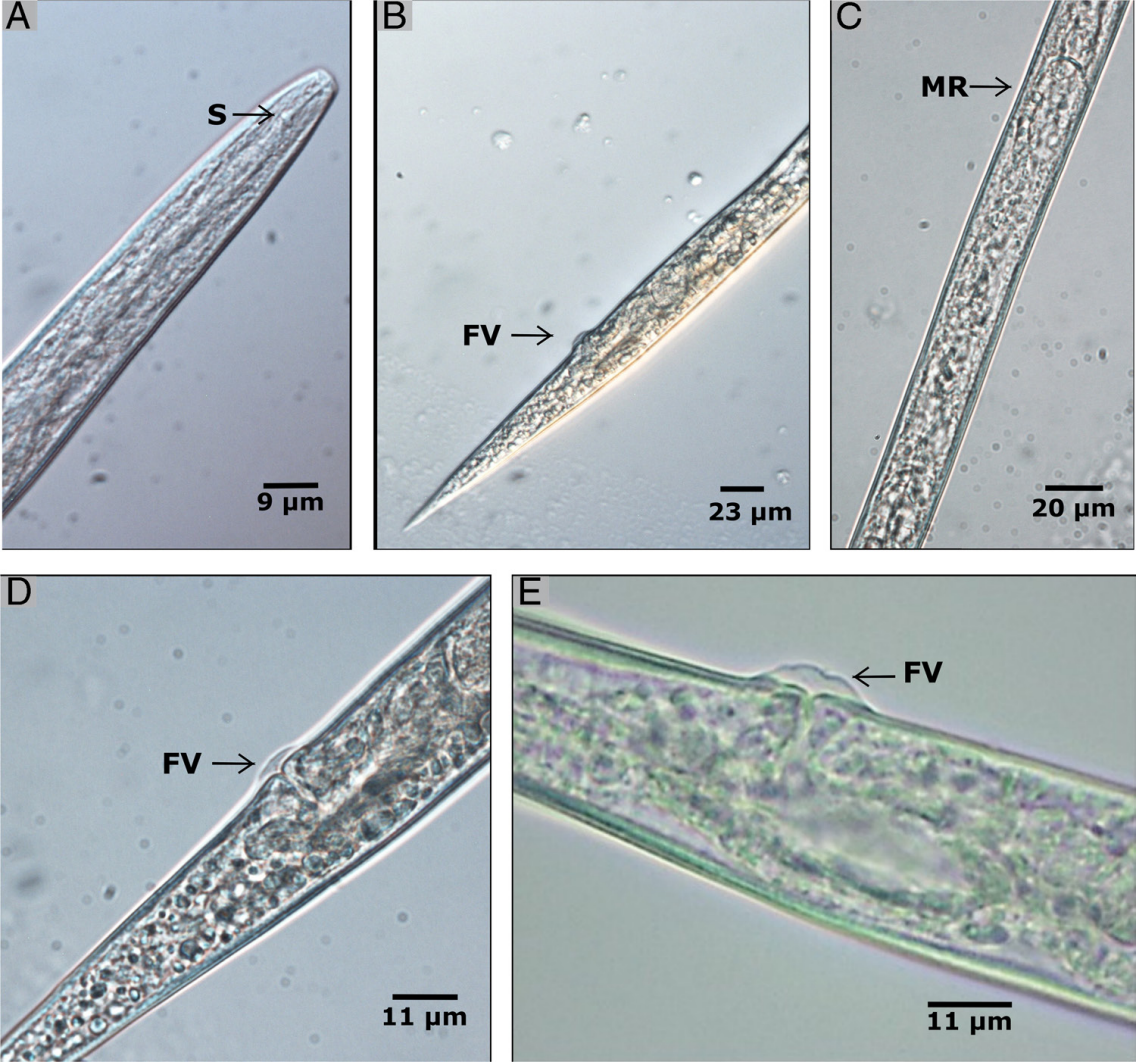
*Pterotylenchus cecidogenus* microphotographs. (A) Anterior region of the female. (B) Posterior region of the female. (C) Middle region of the female. (D, E) Vulval region of the female. S = stylet, FV = Vulval flaps and MR = Middle region.

The nematodes extracted from *D. ovalifolium* stems with gall symptoms presented morphological and morphometric characteristics similar to those reported for the nematode *P. cecidogenus*. Females were morphologically distinguished by the following features: a post-mortem habitus that was straight or slightly ventrally arcuate, a vulva covered by large flaps, and a that was tail elongate-conoid to a sharply pointed tip; no males were identified (Table 2; Fig. 2).

### Molecular characterization and phylogenetic analysis

The amplification of the segment D2-D3 and ITS from the ribosomal DNA region yielded amplicons of 728 and 1000 bp, respectively. The comparison of the sequences against the GenBank database did not present percentages of similarity equal to or greater than 99% with other reference sequences previously reported. However, the sequences of segment D2-D3 were very similar to those of KT192617 and KT192618 (identity levels of 81.06% and 81.14%, respectively; E-value: 0.0) of the species *Ditylenchus phyllobius* (Sinm. *Orrina phyllobia*). Similar results were obtained with the partial sequences of ITS, with 88% similarity to *D. phyllobius* (KT192616.1; E-value of 0.0). The partial sequences obtained in this study are the first reported for *P. cecidogenus* and were deposited for consultation in the NCBI database (Table 3).

**Figure 3:**
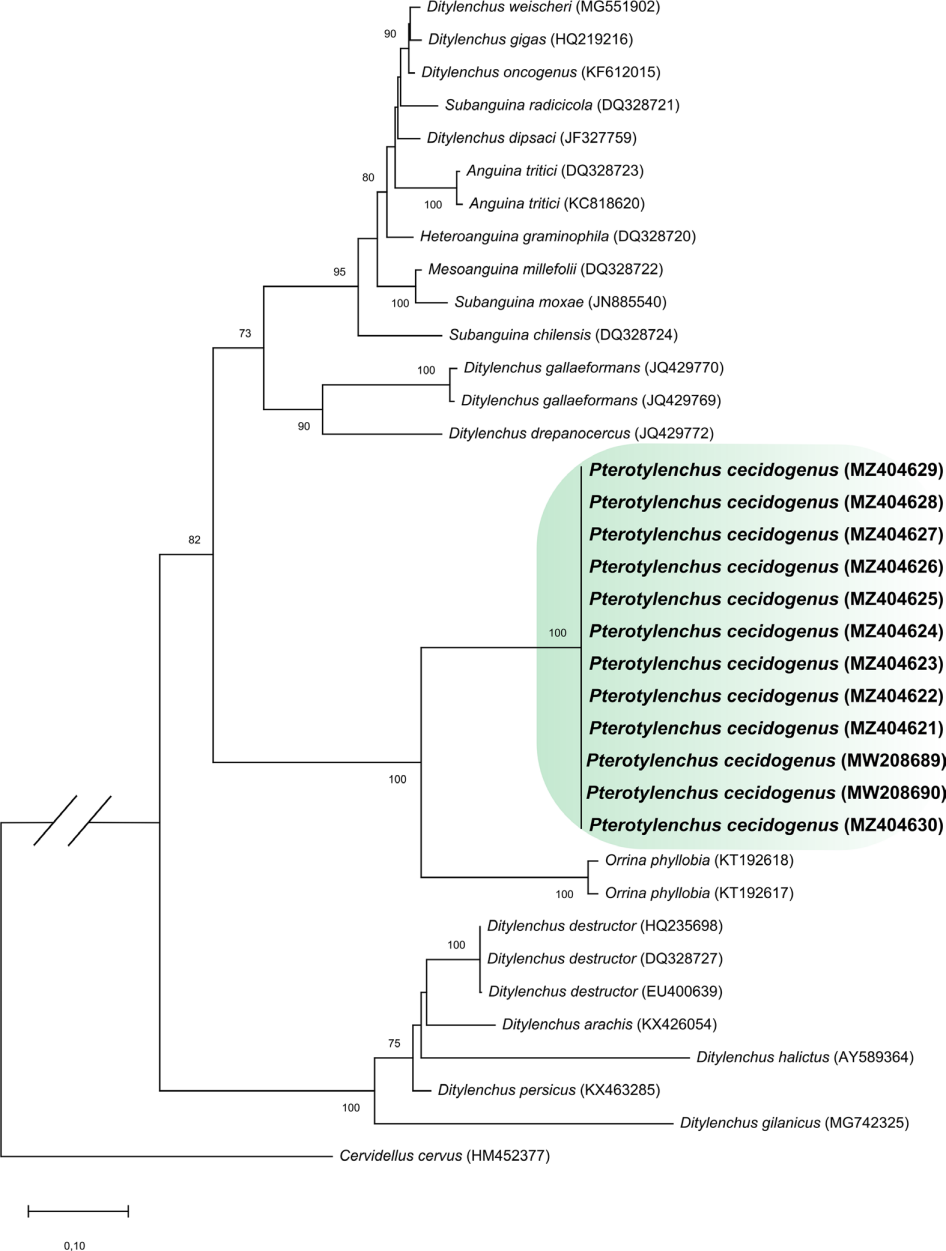
Phylogenetic tree obtained by the statistical method of maximum likelihood based on the Tamura-Nei model of the consensus sequences of the D2–D3 partial segment of *Pterotylenchus* and related genera. The sequences of this work are indicated in bold. The numbers on the nodes indicate bootsrap values of ≥ 70%. The species *Cervidellus cervus* (HM452377) is included as an outgroup.

**Figure 4:**
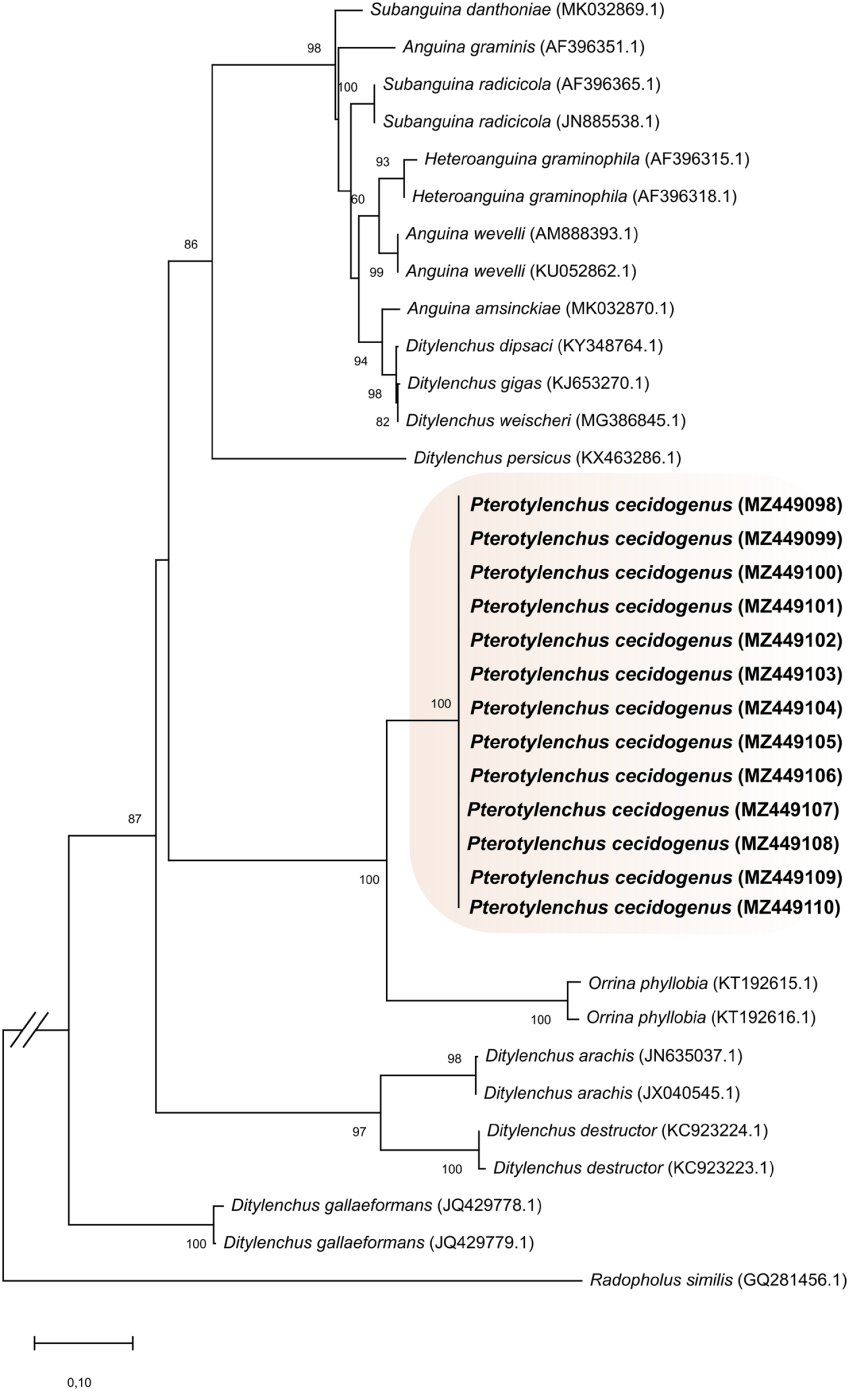
Phylogenetic tree obtained by the statistical method of maximum likelihood based on the Tamura-Nei model of the consensus sequences of the internal transcribed spacer (ITS) of *Pterotylenchus* and related genera. The sequences of this work are indicated in bold. The numbers on the nodes indicate bootstrap values of ≥ 70%. The species *Radopholus similis* (GQ281456) is included as an outgroup.

Phylogenetic analysis based on the use of segment D2-D3 comprised a total of 36 taxa and 824 characters, including gaps, of which 166 were conserved, 636 were variable, and 363 were informative parsimonious sites. Phylogenetic analysis based on the ITS region included 35 taxa and 1499 characters, including gaps, of which 717 were conserved, 736 were variable, and 645 were informative parsimonious sites. In both analyses, the maximum likelihood algorithm grouped *P. cecidogenus* consensus sequences of this study with high bootstrap values of 100%, in a clade separated from other species of the Anguinidae family but close to the clade of *Ditylenchus phyllobius* (Sinm. *Orrina phyllobia*) with 100% bootstrap support (Figs. 3, 4).

### Quantification and analysis of nematode populations

The juvenile stages and females of *P. cecidogenus* were recovered in 56.7% of the tissue samples with galls, with an average population of 1,768 individuals per gram of fresh tissue. In addition, the juvenile and female stages were present in 10% of the node samples, with an average population of 23.8 individuals per gram of fresh tissue. *Pterotylenchus* was not detected in leaf samples. Only three inflorescences were analyzed because our sampling period did not match the flowering season.

Analysis of the parasitic and ecological parameters in roots showed that the genera of parasitic nematodes of plants with the highest relative importance in *Desmodium* roots were *Meloidogyne* (second-stage juveniles) followed by *Pterotylenchus*, *Helicotylenchus,* and *Pratylenchus (*juveniles and females). *Xiphinema* is a plant-parasitic nematode that is less important (Table 4). In relation to other genera, *Pterotylenchus* presented a prominence value of 46.7, with a low distribution (13.3) and population level (12), and eight individuals per gram of fresh root. Differences in the genera *Pterotylenchus* were associated with rhizospheric soil and *Desmodium* roots; however, their parasitic activity was not determined.

**Table 4.** Parasitic and ecological parameters of nematodes associated with *Desmodium ovalifolium* roots.

Nematode	Absolute frequency	Relative frequency	Absolute density	Relative density	Prominence value	Relative prominence value
*Meloidogyne*	33,3	30,2	8,7	25,2	50,2	30,5
*Pterotylenchus*	13,3	12,1	12,8	37,1	46,7	28,3
*Helicotylenchus*	30	27,3	6,2	18	34	20,6
*Pratylenchus*	26,7	24,3	6,3	18,3	32,6	19,8
*Xiphinema*	6,7	5,9	0,5	1,4	1,3	0,76

Note: 30 samples (1 g fresh root).

In the rhizospheric soil of *Desmodium*, the genera of parasitic nematodes of plants of greater relative importance were *Helicotylenchus*, followed by *Tylenchorhynchus (*juveniles and females). *Xiphinema* (juveniles), *Meloidogyne* (second-stage juveniles), and *Pterotylenchus (*juveniles and females) were of intermediate importance. *Pratylenchus (*juveniles and females), *Criconemella,* and *Trichodorus (*juveniles) were recorded in the soil samples, but their distribution and population levels were low (Table 5). *Pterotylenchus* had a prominence value of 11.1, with a low distribution (10) and population level (3.5 individuals per 100 cc of soil).

**Table 5.** Parasitic and ecological parameters of nematodes in rhizospheric soil of *Desmodium ovalifolium.*

Nematode genus	Absolute frequency	Relative frequency	Absolute density	Relative density	Prominence value	Relative prominence value
*Helicotylenchus*	33.3	28,6	32,7	47,6	188,7	64,4
*Tylenchorhynchus*	23.3	20	14,5	21,1	70	23,9
*Xiphinema*	20	17,2	10,5	15,3	46,96	16
*Meloidogyne*	13,3	11,4	4	5,8	14,6	4,98
*Pterotylenchus*	10	8,6	3,5	5,1	11,1	3,8
*Pratylenchus*	6,7	5,7	1,5	2,2	3,9	1,33
*Criconemella*	6,7	5,7	1,3	1,9	3,4	1,16
*Trichodorus*	3,3	2,8	0,7	1	1,3	0,44

Note: 30 samples (100 cc of soil).

## Discussion

The symptoms observed in diseased plants of *D. ovalifolium* used as a cover crop in oil palm in central Colombia include yellowing, foliar drying, wilting, and plant death, and are similar to the descriptions made in previous research (Lenné, 1983; Siddiqi and Lenné, 1984; Stanton, 1986; Lehman, 1991; Varón et al., 2017).

Based on morphological and morphometric diagnosis, the presence of *P. cecidogenus* was confirmed in the stems of *D. ovalifolium* with gall symptoms. The morphometric measurements registered in this study were similar to those reported for *P. cecidogenus* females in the original description (Siddiqi and Lenné, 1984). According to the results obtained with the BLAST tool and phylogenetic analysis with D2-D3 and ITS sequences, *P. cecidogenus* is a sister species of the leaf-galling nematode *D. phyllobius* (Sinm. *Orrina phyllobia*; Medina et al., 2016). These results are consistent with those of Siddiqi and Lenné (1984), who reported that *P. cecidogenus* is a unique species of the Anguinidae family with vulval flaps, but morphologically similar to *Orrina* in lacking a muscular median esophageal bulb and females that are not obese.

The stem nematode *P. cecidogenus* in *D. ovalifolium* presented low absolute frequency and prominence values because the nematode affects the aerial part of the plant; its occurrence in soil and roots does not indicate that it feeds on the root system. However, its presence is possible because when the plants die, the galls remain in the soil, giving the nematode the possibility of feeding in nearby plants (Lenné, 1983).

The nematode *Pterotylenchus* was not detected in the leaf samples analyzed. It is known that this parasite is a nematode that induces galls in the nodes of diseased plants, which explains the high frequency and population found in the galls. Although there were no visible symptoms in the case of nodes, it is possible that they were initiating infection and the galls had not yet formed. Although *Pterotylenchus* appeared in roots, according to Lenné (1983) and Stanton (1986), the parasitic nematode of *D. ovalifolium* induces galls in the nodes of the plants and is not found in the other organs of the plant.

In conclusion, in this study, we found a new distribution of the stem-gall nematode *P. cecidogenus* affecting *D. ovalifolium* plants in central Colombia. This identification was confirmed using molecular tools and constitutes the first report of this technique for this species. This study confirms the spread of the nematode to new regions of the country. This could reflect new movement of plant material or asexual propagation because, since it was first recorded in 1981, it has been restricted to eastern Colombia (Siddiqi and Lenné, 1984). We suggest avoiding the moving plant tissue of *Desmodium* from regions reported with stem-gall nematode *P. cecidogenus* infestations to other areas of Colombia.
